# Evaluate the Sensitivity and Specificity Echocardiography in Trans-Doppler and Tissue Doppler Method in the Estimation of Left Ventricular End-Diastolic Pressure

**DOI:** 10.5539/gjhs.v6n7p92

**Published:** 2014-09-18

**Authors:** M. Hajahmadi Poorrafsanjani, B. Rahimi Darabad

**Affiliations:** 1 Urmia University of Medical Science, Urmia, Iran

**Keywords:** Diastolic function, Doppler, echocardiography, sensitivity

## Abstract

**Background:**

Non-invasive survey of left ventricular end-diastolic pressure (LVEDP) by transmitral Doppler echocardiography and tissue Doppler imaging carries important information about left ventricular diastolic function in chosen subsets of patients. This study is planned to assess whether mitral annular velocities (lateral annulus) as assessed by tissue Doppler imaging and transmitral Doppler echocardiography are associated with invasive measures of left ventricular end diastolic pressure and also the estimation of sensitivity and specificity of these methods.

**Methods:**

One hundred ten consecutive patients admitted to cardiac catheterization underwent simultaneous Doppler interrogation measurements of left ventricular pressure were obtained with fluid-filled pressure. The E/Ea ratio associated well with LVEDP (P<0.005 r=0.4) and the correlation more marked in the patients with reduced contractile function. This correlation was independent of gender.

**Results:**

The E/Ea ration of <8 best discriminated elevated (LVEDP>12) from normal LVEDP with a sensitivity of 73.5% and specificity 57.8%, PPV and NPV were 75.75% and 55% respectively. Our study results also showed that quantitative estimation of LVEDP could be suggested by the equation of LVEDP=1.2 E/Ea+6.67± 8 mmHg P<0.005 B=0.4.

Male-LVEDP=0.9 E/Ea + 7.78±7.67 mmHg (r=0.4 Pa<0.005)

EF>_50% -+LVEDP=1.48 E/Ea + 9.05±5.23 (r=0.4 P<0.05)

EF<50% -+LVEDP=0.76 E/Ea + 8.4±2.3 (r=0.5 P<0.005)

## 1. Introduction

Heart failure is a major problem of all types of heart disease. Heart failure is a relatively common disease and it is estimated that 4/6 million people in America are undergoing treatment and in a year, 55 thousand new cases are added. The prevalence of heart failure increases significantly with age, and about 1 to 2 percent of people aged 59-50 years and over 75 to 10% are common. The final diagnosis at discharge in patients with heart failure in the United States is over 65 years. Despite a steady decline in the incidence of coronary heart disease and stroke, the incidence and prevalence of heart failure is increasing. From 1985 to 1995 the number of admissions to hospital for heart failure increased by 51% and 870 thousand hospital discharge occurred in 1996 due to heart failure (Husain et al., 2013). In America, almost 45 thousand deaths occurred each year due to heart failure in addition, heart failure is an associated cause of 250 thousand other deaths. This increase is partly due to the increasing the age of people and partly due to the improved survival of patients with coronary artery disease. Several economic impact of heart failure on health in America has caused disability and impairment costs of medical treatment and diagnostic disorder (Sattarzadeh et al., 2014). Health expenditures in 1994 amounted to $38 million has been estimated that 23 million was due to hospitalization. Costs of hospitalization in patients with heart failure have twice the spending of all types of cancers and myocardial infarction (Braunwald, 2012). Physiological definition of heart failure can be caused due to systolic dysfunction, leading to impaired blood out (systolic failure) or abnormality in ventricular diastolic function causing disturbances in the filling (diastolic dysfunction). Systolic heart failure is more attuned and in fact classic heart failure is correlated to impaired inotropic stage. What is less familiar but equally important, diastolic heart failure in which the heart’s functioning to accept blood is impaired which may be caused by reduced or incomplete ventricular relaxation. This type of failure may be transient, such as the cases of ischemic or permanent, such as concentric hypertrophy or secondary bordered cardiomyopathy to infiltrative disorders such as amyloidosis. Epidemiological studies have shown that diastolic heart failure is more common than what previously mentioned. Especially is common in older women with hypertension. However, systolic or diastolic dysfunction is simultaneous in many patients. The most common form of heart failure caused by chronic coronary artery ailment is an example of systolic and diastolic dysfunction together. In this situation systolic dysfunction arising from chronic reduction of secondary myocardial contractility to prior MI and secondary reduced myocardial contractility to temporary ischemia.

Approximately one third of patients with CHF whose diastolic failure is prevail that as pulmonary or systemic congestion and complications caused by the presence of normal or near-normal systolic function, another one third, have combined systolic and diastolic dysfunction. Others one third have only systolic dysfunction. In the Framingham study population, patients who have CHF 51% of them had EF about 50% or higher-65% of those who had EF of more than or equal 50% were women, but 75% of patients who had EF less than 50% were men- mortality in patients who had EF of more than or equal 50% was 8.7%, compared to patients with EF below 50% (about 18.9% mortality) is low but is about 4 times higher than the control group that in terms of age are match. In the study of Olmstand County/Minnesota similarly poor prognosis of diastolic and systolic dysfunction has been reported. Diastolic dysfunction often happens in the absence of systolic dysfunction. This disorder can be caused by increased myocardial stiffness or impaired relaxation that the latter leads to a decrease in end-diastolic dilation. When this happens, the pressure of the dilated left ventricle rises to achieve the same end-diastolic volume that result in increased venous pressure and pulmonary congestion takes place (Braunwald, 2012; Oho & Jae, 1999). Intra-cardiac pressure measurements are important variables that are used in the evaluation of cardiac function and estimation of left ventricular filling pressure obtained. These pressures are measured partially through a fluid-filled catheter. This technique can create artifact that may be invasive-requiring fluoroscopy and equipment with a special center and cannot be easily done. Recently, Doppler echocardiography techniques with non-invasive method assess the pressures within the heart (Braunwald, 2012). The need for non-invasive methods for the assessment of left ventricular filling by echocardiography is answered. Evaluation of mitral flow by Doppler echocardiography in past 15 years was original clinical and non-invasive method for the assessment of diastolic function, which specifies different forms of left ventricular filling. Factors affecting the mitral flow include: location of the sample, breathing-heart beating-cardiac conduction disturbances-patient’s age. Tissue Doppler is a new technique and recommended for the diastolic function with straight up mitral annulus motion and its importance is in the assessment of left ventricular filling and relatively free of the influence of pre-loaded variations. Differential diagnosis of diastolic dysfunction in patients in early stage (pseudo normalization) with a sensitivity of 88% from normalized cases and specificity of 67% makes possible (Catherin & Otto, 2002). Measurement of left ventricular occupied with echocardiography compared with invasive methods is:

1-Does not require dye or radio Nuclides.
2-Will not be exposed to x-rays.
3-It is more secure and safe.
4-Repeated measurements are possible.
5-Does not need for special place equipment.
6-Does not cause hemodynamic changes.
7-Relatively easy.
8-It is reliable.
9-Gives immediate information.
10-Show common changes of the heart (Arthur, 1994).

Doppler echocardiography can cause problems that include: 1-Good video quality was not possible in some patients 2-Techniques related to the performer 3 – With changing of the location the sample is changed. Therefore, echocardiography should be performed by trained personnel carefully and diligently (Arthur, 1994; Isaaz, Thompson et al., 1989; Isaaz, Munos del Romeral et al., 1993). This study aimed to replace invasive methods with higher sensitivity and accuracy, the accuracy of tissue Doppler imaging in the estimation of left ventricular filling pressures in detection of cases with impaired cardiac diastolic and estimation of left ventricular filling pressures based on heart systolic performance compare with each other.

## 2. Assessment of Cardiac Function

Diastolic function indices can be classified into three groups.

1-Isovolemic Relaxation criteria.
2-Indices of left ventricular diastolic pressure that is obtained from the pressure-volume relation.
3-The form of ventricular diastolic filling is obtained by Doppler echocardiography, radionuclide angiography.

Note that since a similar study has not been done and the aforementioned study has been done in subjects with different ages and in different sample volumes without sex comparison and sometimes uncertain results of PCWP associated with calculations of non-invasive or trans-mitral Doppler echocardiography and tissue Doppler is obtained. We are to do research in the field of coronary risk factors, presence or absence of coronary failure in patients with different ages and type of left ventricular end-diastolic pressure estimation with non-invasive methods and compare this calculation in patients with normal and abnormal cardiac systolic function.

## 3. Methods

In this study that is an observational study (descriptive-analytic) 110 patients are examined with non-random and consecutive sampling method. People who were candidates for coronary angiography and mild cardiac valve disease were enrolled after obtaining informed consent from them. After the selection of samples and informed consent by patients, patients were transferred to lab coat and end-diastolic left ventricular pressure by fluid-filled catheter connected to a transducer method was measured by digital catheter in the left ventricle. Transducer zero parallel to the mid-axillary line was set before the angiography catheter and route of the catheters to transducer was checked in terms of the absence of air bubbles. Left ventricular end-diastolic pressure curve in 5 end-breath cycles were recorded. Left ventricular pressure curve record had done under the trained technician (collaborator), then left ventricular end-diastolic pressure by collaborator in c-point by line drawing of the R-wave in EKG simultaneously, was calculated by collaborator and form 1 was completed. A day after angiography (at least 12 hours passed from angiography) the patient was referred to the center of angiography and echocardiography were performed by collaborator with vigmed system 5. First the left ventricular function was calculated by Simpson’s method in Vivian 4C and then trans-mitral flow measured with beam position transducer at the tip of the mitral leaflets with a maximum angle of 20 from the flow path with pulse Doppler amount of E-5 end-expiratory cycles in terms of m/sec was calculated and then system was regulated to investigate tissue Doppler and lateral mitral annulus motion in sample size 1.5-2mm with the proper sweep and speed of 50-25, the amount of Ea. In terms of m/sec was calculated and at the end form 2 has been completed. Note that the person performing echocardiography during echocardiography was not informed of angiography result and the amount of specified pressure in lab coat, then the raw tables were completed and the ratio of E/Ea and its relation to left ventricular end-diastolic pressure was measured. Also exclusion criteria are as follows:

1-Moderate to severe valve disease
2- Lack of patient corporation
3- Hemodynamic instability of patient for referral to echocardiography.

At the end SPSS software was used to data analysis of the study (Kaveh et al., 2014).

## 4. Results

Of 110 patients, 38 (34.55%) were female and 72 (65.45%) were male and the age range was (78-35 years) that 76 (69.1%) were under 65 years and 34 (30.90%) were equal to or greater than 65 years. According to coronary risk factors and extent of coronary artery disease patients in two groups with increased left ventricular end-diastolic pressure (patients with diastolic heart failure) and those with normal end-diastolic pressure LVEDP ≤ 12mHg are shown in Tables 2 and 3. (According to the present study women have more coronary risk factors than men, but history of MI and SM is less).

**Table 1:** Stages of diastolic dysfunction

parmeter	normal (young)	normal (adult)	delayed relaxation	pseudo-normal filling	restrictive filling
E/A (CM/SEC)	>1	>1	<1	1-2	>2
DT (MSEC)	<200	<220	>220	150-200	<150
IVRT (MSEC)	<100	<100	>100	60-100	<60
S/D	<1	≥1	≥1	<1	<1
AR (CM/SEC)	<35	<35	<35	≥35*	≥25*
V_P_ (CM/SEC)	>55	>45	<45	<45	<45
E_A_ (CM/SEC)	>10	>8	<8	<8	<8


Unless atrial mechicaI failure is present.

AR=pulmonary venous peak atrial contraction reversed velocity DT=early left venlricular filling deceleration time; E/A=earl*to* atrial left ventricular filling ratio; E_A_=peak early diastolic annular myocardial velocity; IVRT=isovolumic relaxation lime: S/D=systolic-to-diastolic pulmonary

Venous flow ratio; V_p_=color M-mode flow propagation vekcity.

From Garciamj et al. New Doppler echocardiographic applicatoins for the study of diastolicfunction (J Am coll Cardial 32:872, 1998).

**Table 2:** Comparison of different risk factors based on diastolic heart failure

Rick factor		LVEDP>12	LVEDP=<12
HTN	-	68.1%(49)	71.1%(27)

	+	31.9%(2)	28.9%(1)

HLP	-	76.4%(55)	76.3%(29)

	+	23.6%(17)	23.7%(9)

DM	-	73.6%(53)	71.1%(27)

	+	26.4%(19)	28.9%(11)

SM	-	72.2%(52)	71.1%(27)

	+	27.8%(20)	28.9%(11)

Obesity	-	72.2%(52)	73.7%(28)

	+	27.8%(2)	26.3%(1)

MI	-	73.6%(53)	76.3%(29)

	+	26.4%(19)	23.7%(9)

SEX	F	31.9%(23)	39.5%(15)

	M	68.1%(49)	60.5%(23)

AGE	<65y	68.1%(49)	71.1%(27)

	=>65y	31.9%(23)	28.9%(11)

**Table 3:** Comparison of coronary artery disease based on diastolic heart failure

	LVEDP>12	LVEDP=<12
NL	16.7%(12)	26.3%(10)
1VD	13.9%(10)	15.8%(6)
2VD	16.7%(12)	18.4%(7)
3VD	52.8%(38)	39.5%(15)
TOTAL	100%(72)	100%(38)

Unless atrial mechicaI failure is present.

AR=pulmonary venous peak atrial contraction reversed velocity DT=early left venlricular filling deceleration time; E/A=earl*to* atrial left ventricular filling ratio; EA=peak early diastolic annular myocardial velocity; IVRT=isovolumic relaxation lime: S/D=systolic-to-diastolic pulmonary

Venous flow ratio; Vp=color M-mode flow propagation vekcity.

From Garciamj et al. New Doppler echocardiographic applicatoins for the study of diastolicfunction (J Am coll Cardial 32:872, 1998).

As is evident from Table 2 diastolic dysfunction in patients, percentage of hypertension risk factors-obesity-history of heart attack than those with normal diastolic function was higher. Also, people with diastolic dysfunction were older than the other group (31.99 vs. 28.99%). Table 3 provides the distribution of coronary artery disease, patients with LVEDP>12 percentage of 3VD was more than those with LVEDP≤12 (52.8 vs. 39.51%) and the percentage of normal coronary artery in patients with normal diastolic function is more (26.3% compared to 167% in patients with diastolic dysfunction). Comparison of diastolic dysfunction and its association with cardiac systolic function is shown in Table 4. As is evident 30% of the subjects had normal systolic and diastolic function and 39.06% of the subjects had only diastolic dysfunction and their systolic function was preserved.

**Table 4:** Comparison of diastolic and systolic heart failure

COUNT	EF<50%	EF=>50%	TOTAL
LVEDP=<12	5(4.54%)	33(30%)	38(34.54%)
LVEDP>12	29(26.36%)	43(39.09%)	72(65.45%)
TOTAL	34(30.9%)	76(69.09%)	110(100%)

## 5. Discussion

The statistical tests used for quantitative variables from central indexes (median-mode-mean) and dispersion index of SD and for comparison of E/Ea or end-diastolic pressure Pearson’s correlation coefficient logistic regression was used. The statistical analysis was performed using SPSS, then curve was drawn and then with drawing a regression curve ventricular end-diastolic pressure was calculated with the formula:

LVEDP=1.2E/Ea +6.67 ± 8mHg P <0.005 r=0.4

Left ventricular end-diastolic pressure is accessed in terms of sex using the following formula:

Male→LVEDP=1.38E/E_a_+6.06±8.07mHg r=0.4 p<0.005

Female →LVEDP=0.9E/E_a_+7.78±7.67mHg r=0.4 P<0.005

Regression curve based on left ventricular function with two groups of normal systolic function of the heart (preserved) using the following formula to diagnose their diastolic function.

LVEDP=1.48E/E_a_+9.05±5.23mHg (r=0.4, p<0.05)

People with impaired diastolic and systolic function in combination

LVEDP=0.76E/E_a_+8.4±2.3mHg (r=0.5, p<0.005)

As would be expected in patients with abnormal systolic function, measurement of left ventricular end-diastolic pressure with echocardiography was more accurate than those with normal systolic function. Cut of point ratio E/Ea according to regression curves were calculated by the number 8. The proportion of patients with E/Ea less than 8 are non-diastolic dysfunction and in those with E/Ea equal to or greater than 8, are with diastolic heart failure. According to Table 5, echocardiography was performed by Doppler trans-mitral and tissue Doppler method with determining the ratio of E/Ea ¬ to calculate the left ventricular end-diastolic pressure, have sensitivity equal to 73.5% and specificity equal to 57.8% and PPV and NPV was calculated 75.75% and 55%, respectively. Limitations of this study include translational and rotational motions of the mitral annulus, which can have influence on the interpretation of diastolic dysfunction, it is noteworthy that in similar studies the amount of it has considered slight and with careful and correct determination of the sampling location by the person performing it and use of annulus lateral instead of septal the error is minimal. The effect of WMA on the motion of mitral annulus in similar studies had no significant impact. But it needs to be investigated further in future studies. Differentiation of different forms of heart failure especially normal diastolic function of the type of pseudo-normalization in trans-Doppler this technique (TDI) may help. This study was estimation of left ventricular end-diastolic pressure with non-invasive method independent of sex, and in both sexes calculation of the LVEDP was statistically significant and not different. As the calculation of the LVEDP in cases with systolic heart failure was more accurate, but the correlation between E/Ea and LVEDP in both groups of cases with systolic heart failure and cases with preserved systolic function was statistically significant. Given that previous studies on the assessing of left ventricular filling pressures by Doppler trans-mitral only in those with systolic dysfunction of heart was not statistically significant, raise the clinical value of this study. According to sensitivity and acceptable PPV this method and results of previous studies (sensitivity 73.5% vs. 81%) (Isaaz Munos del Romeral et al., 1993) Echocardiography with trans-Doppler and tissue Doppler method can be replaced as non-invasive estimation of left ventricular end-diastolic pressure with invasive methods and with its aid benefit in the diagnosis of heart failure-treatment options for patients-patient follow-up and response to treatment.
